# A French National Real-World Survey of People With Multiresistant HIV-1 Viruses Receiving an Antiretroviral Regimen Including Fostemsavir or Ibalizumab

**DOI:** 10.1093/ofid/ofag495

**Published:** 2026-08-14

**Authors:** Karl Stefic, Camille Tumiotto, Anne de Monte, Marc Wirden, Audrey Rodallec, Djeneba Fofana, Marie-Laure Chaix, Pantxika Bellecave, Elisabeth Garnier, Justine Sourice, Camille Vellas, Stéphanie Raymond, Sidonie Lambert-Niclot, Enagnon Kazali Alidjinou, Pauline Coulon, Véronique Avettand-Fenoel, Gilbert Mchantaf, Lynda Handala, Elodie Alessandri-Gradt, Alice Moisan, Minh Lê, Constance Delaugerre, Anne-Geneviève Marcelin, Gilles Peytavin, Vincent Calvez, Diane Descamps, Charlotte Charpentier, the Entry Inhibitors Observatory Study Group, Kevin Alexandre, Clotilde Allavena, Antoine Bachelard, Fabrice Bonnet, Charles Cazanave, David Chirio, Yoann Conan, Eric Cua, Pierre Delobel, Jade Ghosn, Laurent Hocqueloux, Karine Lacombe, Estibalitz Lazaro, Adrien Lemaignen, Guillaume Martin-Blondel, Jean-Michel Molina, Bao Phung, Valérie Pourcher, Caroline Proux, Pascal Pugliese, Mayda Rahi, Christophe Rioux, Olivier Robineau

**Affiliations:** Université de Tours, INSERM 1259 MAVIVHe, CHU de Tours, Service de Virologie, Tours, France; CHU de Bordeaux, Service de Virologie, CNRS-UMR 5234 Microbiologie Fondamentale et Pathogénicité, Université de Bordeaux, Bordeaux, France; CHU de Nice, Service de Virologie, Nice, France; Sorbonne Université, INSERM, UMR-S 1136, Institut Pierre Louis D’Epidémiologie et de Santé Publique, AP-HP, Hôpitaux Universitaires Pitié Salpêtrière–Charles Foix, Service de Virologie, Paris, France; CHU Nantes, Service de Virologie, Nantes, France; Santé Sorbonne Université, INSERM, Institut Pierre Louis D’Epidémiologie et de Santé Publique APHP, Hôpital Saint-Antoine, Département de Virologie, Paris, France; Service de Virologie, INSERM U941, AP-HP, Hôpital Saint-Louis, Université Paris Cité, Paris, France; CHU de Bordeaux, Service de Virologie, CNRS-UMR 5234 Microbiologie Fondamentale et Pathogénicité, Université de Bordeaux, Bordeaux, France; CHU Nantes, Service de Virologie, Nantes, France; CHU Nantes, Service de Virologie, Nantes, France; INSERM UMR 1291, CHU Toulouse Purpan, Service de Virologie, Toulouse, France; INSERM UMR 1291, CHU Toulouse Purpan, Service de Virologie, Toulouse, France; Santé Sorbonne Université, INSERM, Institut Pierre Louis D’Epidémiologie et de Santé Publique APHP, Hôpital Saint-Antoine, Département de Virologie, Paris, France; Univ Lille, CHU de Lille, Laboratoire de Virologie ULR3610, Lille, France; Univ Lille, CHU de Lille, Laboratoire de Virologie ULR3610, Lille, France; Université d’Orléans, LI2RSO, CHU d’Orléans, Service de Virologie, Orléans, France; Université d’Orléans, LI2RSO, CHU d’Orléans, Service de Virologie, Orléans, France; Université de Tours, INSERM 1259 MAVIVHe, CHU de Tours, Service de Virologie, Tours, France; Univ Rouen Normandie, Université de Caen Normandie, INSERM, Normandie Univ, DYNAMICURE UMR 11, CHU Rouen, Department of Virology, National Reference Center of HIV, Rouen, France; Univ Rouen Normandie, Université de Caen Normandie, INSERM, Normandie Univ, DYNAMICURE UMR 11, CHU Rouen, Department of Virology, National Reference Center of HIV, Rouen, France; Service de Pharmacologie, AP-HP, Hôpital Bichat-Claude Bernard, Université Paris Cité, INSERM, IAME, Paris, France; Service de Virologie, INSERM U941, AP-HP, Hôpital Saint-Louis, Université Paris Cité, Paris, France; Sorbonne Université, INSERM, UMR-S 1136, Institut Pierre Louis D’Epidémiologie et de Santé Publique, AP-HP, Hôpitaux Universitaires Pitié Salpêtrière–Charles Foix, Service de Virologie, Paris, France; Service de Pharmacologie, AP-HP, Hôpital Bichat-Claude Bernard, Université Paris Cité, INSERM, IAME, Paris, France; Sorbonne Université, INSERM, UMR-S 1136, Institut Pierre Louis D’Epidémiologie et de Santé Publique, AP-HP, Hôpitaux Universitaires Pitié Salpêtrière–Charles Foix, Service de Virologie, Paris, France; Service de Virologie, AP-HP, Hôpital Bichat-Claude Bernard, Université Paris Cité, INSERM, IAME, Paris, France; Service de Virologie, AP-HP, Hôpital Bichat-Claude Bernard, Université Paris Cité, INSERM, IAME, Paris, France

**Keywords:** fostemsavir, HIV, ibalizumab, pharmacology, resistance

## Abstract

**Background:**

Attachment inhibitor fostemsavir (FTR) and postattachment inhibitor ibalizumab (IBA) are used in people with HIV-1 (PWH) who are highly treatment experienced and harboring multidrug-resistant viruses, and real-world data remain limited. We describe population characteristics and pharmacovirologic outcomes of PWH initiating FTR- or IBA-based regimens.

**Methods:**

We conducted a French retrospective observational study within the AIDS Research National Agency | Emerging Infectious Diseases virology and pharmacology network. Virologic failure (VF) was defined as 2 consecutive plasma viral loads (VLs) ≥50 copies/mL; nonvirologic response was considered a VL decrease <1 log_10_ copies/mL or failure to achieve virologic suppression.

**Results:**

Among 70 PWH receiving FTR-based treatment, 50% were virologically suppressed at initiation; median VL among viremic cases was 3.6 log_10_ copies/mL. Resistance to at least 2 antiretrovirals was observed among participants by inhibitor class: 96%, nucleoside reverse transcriptase inhibitor; 94%, nonnucleoside reverse transcriptase inhibitor; 74%, protease inhibitor; and 64%, integrase strand transfer inhibitor. The genotypic susceptibility score was ≤1 in 47%, and median follow-up was 20 months. Eight VFs and 8 nonvirologic responses occurred. At failure, emergence of FTR resistance-associated mutations occurred in 1 case. Suboptimal temsavir concentrations were observed in 2 of 8 participants in failure. Eleven PWH who were viremic initiated IBA-based treatment: the genotypic susceptibility score was ≤1 in 4, and median follow-up was 7 months with 5 VFs and 2 nonvirologic responses. One new resistance mutation (N74D, capsid) was detected at VF; suboptimal plasma concentrations of oral antiretrovirals were observed in 3 of 5 participants.

**Conclusions:**

PWH receiving FTR or IBA had extensive multidrug resistance and limited therapeutic options. Among PWH who were viremic and initiating FTR- and IBA-based regimens, virologic success was achieved in 63% and 36%, respectively, and maintained in 91% who were virologically suppressed and starting an FTR-based regimen.

## Teaser

This observational study examined fostemsavir and ibalizumab use in real-life settings. Among participants who were highly treatment experienced and viremic and had multidrug-resistant viruses, 63% and 36% reached virologic suppression (<50 copies/mL) when initiating fostemsavir- and ibalizumab-based regimens, respectively.

The long-term success of modern combination antiretroviral (ARV) therapy has transformed HIV-1 infection into a manageable chronic disease. However, virologic failure (VF) due to cumulative drug resistance remains a major clinical challenge in people with HIV-1 (PWH) who are heavily treatment experienced (HTE). Depending on studies and definitions, individuals who are HTE can represent up to 10% of PWH in Western countries and can have limited therapeutic options with extensive resistance across multiple ARV classes [1–5]. Recent reports showed increasing resistance to second-generation integrase strand transfer inhibitors (INSTIs) in several African cohorts, which could lead to the emergence of a higher proportion of multidrug-resistant (MDR) viruses in the future [6, 7]. Consequently, there has been sustained interest in the development of new ARV agents with alternative mechanisms of action that retain activity against MDR HIV-1 and exhibit minimal cross-resistance with existing drug classes. Fostemsavir (FTR), ibalizumab (IBA), and more recently lenacapavir (LEN) are the latest drugs having received approval for HIV treatment, each representing a new drug class with a distinct mode of action [8–13].

FTR is the first approved representative of the HIV-1 attachment inhibitor class. It is a phosphonooxymethyl prodrug of temsavir, targeting the HIV-1 envelope glycoprotein gp120. It binds to a conserved pocket proximal to the CD4 binding site. This interaction stabilizes gp120 in a prefusion conformation that prevents engagement with the CD4 receptor on host CD4+ T cells, thereby blocking the initial attachment step required for viral entry [14, 15]. The clinical efficacy of FTR has been most extensively evaluated in HTE populations with MDR HIV-1. The pivotal phase 3 BRIGHTE study enrolled participants with limited or no remaining fully active ARV options and demonstrated that FTR, when combined with optimized background therapy (OBT), provided sustained virologic and immunologic benefit [16, 17]. At 48 and 96 weeks, a substantial proportion of participants achieved HIV-1 RNA suppression, accompanied by meaningful increases in CD4+ T-cell counts. In vitro studies have demonstrated that temsavir exhibits antiviral activity across a broad range of HIV-1 subtypes, although intrinsic susceptibility varies among isolates due to sequence heterogeneity within gp120 [14]. Thus, FTR is not active on CRF01_AE, HIV-1 group O, and HIV-2 [14]. Substitutions at positions S375, M426, M434, and M475 can be detected in viruses from individuals experiencing VF on FTR-containing regimens [14, 18]. In the BRIGHTE study, several participants developed gp120 substitutions associated with reduced temsavir susceptibility; however, these changes did not uniformly predict treatment failure, and some individuals achieved or maintained virologic suppression despite having viruses with measurable reductions in phenotypic susceptibility [17, 19, 20]. These findings suggest that the outcome of an FTR-containing regimen is still difficult to predict and that treatment failure is not fully understood, reflecting the novelty of the drug and the complexity of gp120-mediated resistance mechanisms.

IBA is a humanized monoclonal antibody and the first-in-class postattachment inhibitor. The antibody targets the host CD4 receptor rather than viral proteins, thereby preventing conformational changes essential for the interaction with the envelope glycoprotein gp120 and for viral entry into T cells [8, 21, 22]. This mechanism distinguishes it from other ARVs that target viral enzymes, proteins, or co-receptors. Its in vitro neutralization activity is broad, exhibiting potent inhibition against diverse HIV-1 isolates [23, 24] and HIV-2 [25]. Phase 3 studies of individuals who were HTE with MDR HIV-1 infection and failing regimens showed a significant viral load (VL) decrease among those receiving IBA combined with an OBT. Approximately half of participants achieved VLs <50 copies/mL by 24 weeks and maintained these levels at 48 weeks [11]. Resistance mechanisms involve alterations in the gp120 envelope glycoprotein, and genotypic prediction is complex, including a combination of factors, such as the number of potential *N*-glycosylation sites in the V5 loop, the V2 loop length, or amino acids at position 375 and 386 of gp120 [23, 26, 27].

Longer-term effectiveness, evaluation of resistance evolution, and optimal resistance testing strategies need to be further investigated for FTR and IBA. However, their use has been limited to a small number of PWH in the context of salvage therapy, and there are few data available on their use in real-world settings. Here, we describe the population characteristics and pharmacovirologic outcome of PWH initiating an FTR- or IBA-based treatment in a national retrospective observational study conducted in France.

## PARTICIPANTS AND METHODS

### Study Participants and Virologic Outcome Definition

A French national retrospective observational study of PWH receiving an FTR- or IBA-based treatment for at least 3 months was conducted within the AIDS Research National Agency | Emerging Infectious Diseases (ANRS|MIE) virology and pharmacology network. All participants had at least 1 prior genotypic resistance test (GRT) and were considered HTE based on resistance criteria—specifically, exhibiting viruses with drug resistance mutations to at least 1 ARV of 2 major drug classes. A VF was defined as 2 consecutive plasma VLs ≥50 copies/mL, and a nonvirologic response was defined as a VL decrease <1 log_10_ copies/mL or failure to achieve virologic suppression (<50 copies/mL) at week 24.

### Ethics

This study was conducted in accordance with the Declaration of Helsinki and was a retrospective noninterventional study with no addition to standard care procedures. All PWH gave their written informed consent to have their medical charts recorded in the electronic medical record system Nadis (Fedialis Medica, Commission Nationale de l’Informatique et des Libertés [CNIL] 770134, 30 October 2001). Reclassification of biological remnants into research material after completion of the ordered virologic tests was approved by the local interventional review board of Pitié-Salpêtrière hospital. Such protocols are exempt from individual informed consent according to the French Public Health Code (CSP Article L.1121–1.1). This study received a declaration of conformity from the CNIL to reference methodology framework MR-004 on 4 November 2024 (reference 2236305), in compliance with the French/European Union requirements of the General Data Protection Regulation. This study has also been declared in the French Health Data Hub (20251013133851).

### Data Collection

Immunovirologic and therapeutic status at baseline was collected. Historical RNA genotypes were collected, and cumulative GRT results were interpreted via the ANRS|MIE algorithm (https://hivfrenchresistance.org). Major protease inhibitor (PI) resistance mutations were defined according to the International Antiviral Society–USA resistance mutations list [28]. Genotypic susceptibility scores (GSSs), with scores of 1 for susceptibility, 0.5 for possible resistance, or 0 for full resistance, were determined by ANRS|MIE algorithm V35 (www.hivfrenchresistance.org). The overall susceptibility score of the OBT was the sum of the individual scores.

Temsavir plasma concentrations and ARV plasma concentrations of the OBT were determined by ultra-performance liquid chromatography–tandem mass spectrometry (Waters Acquity) with a limit of quantification <5 ng/mL. Results were interpreted using a cutoff of 200 ng/mL for temsavir, corresponding to the protein-adjusted 50% effective concentration allowing a change of 1 log_10_ copies/mL in plasma VL after 7 days of monotherapy [29].

## RESULTS

### Description of PWH Initiating FTR- or IBA-Based Treatment

Seventy PWH receiving FTR-based treatment were included between January 2015 and January 2024 in 10 clinical centers. Half (n = 35) were virologically suppressed at baseline (VL <50 copies/mL), and half were in treatment failure with a median VL of 3.6 log_10_ copies/mL (IQR, 2.4–4.5). Overall, the median CD4 cell count at initiation was 419 cells/mm^3^ (IQR, 208–660). The median CD4 cell count at initiation was significantly higher among individuals who were virologically suppressed than among those in treatment failure (585 vs 219 cells/mm^3^; *P* < .0001, Wilcoxon test).

Eleven participants receiving IBA-based regimen were included between 2019 and 2023 in 6 clinical centers. All were in treatment failure at the time of IBA-based treatment initiation with a median VL of 4.3 log_10_ copies/mL (IQR, 2.7–4.8). Participants characteristics are depicted in Table 1. Four participants received both FTR and IBA: 2 received them as part of the same regimen, while the other 2 received them at different times and as part of different regimens. The description and virologic outcomes of these participants are described in the observatories. Many ARV combinations were received by the participants prior to FTR- or IBA-based treatment, the most common being 2 nucleoside reverse transcriptase inhibitors (NRTIs) + boosted PI + INSTI for FTR-based treatment (n = 8, 11%) and 2 NRTIs + INSTI for IBA-based treatment (n = 4, 36%; Table 1).

**Table 1.** Study Participant Characteristics

	FTR (n = 70)			
	VL <50 Copies/mL at BL (n = 35)	VL >50 Copies/mL at BL (n = 35)	All (n = 70)	IBA (n = 11)
Age, y	59 (54–63)	55 (50–61)	57 (53–63)	39 (31–55)
Men	30 (86)	23 (66)	53 (76)	4 (36)
Nadir CD4 cells count,/mm^3^	53 (25–189)	23 (5–115)	51 (9–137)	10 (2–41)
Zenith HIV VL, log_10_ c/mL	5.5 (4.7–5.7)	5.7 (5.1–6.0)	5.6 (4.9–5.9)	5.7 (5.4–6.1)
Viral tropism				
R5	5/18 (28)	5/22 (23)	10/40 (25)	2/6 (33)
X4	13/18 (72)	17/22 (77)	30/40(75)	4/6 (67)
HIV-1 B subtype	32 (91)	29 (83)	61 (87)	5 (45)
HIV-1 VL at initiation, log_10_ copies/mL	…	3.6 (2.4–4.5)	…	4.3 (2.7–4.8)
CD4 cell count at initiation, per mm^3^	585 (406–737)	219 (70–415)	419 (208–660)	70 (45–110)
Prior antiretroviral regimen^a^				
2 NRTI + bPI	1 (3)	3 (9)	4 (6)	3 (27)
2 NRTI + INSTI	2 (6)	5 (14)	7 (10)	4 (36)
2 NRTI + bPI + INSTI	4 (11)	4 (11)	8 (11)	…
NNRTI + bPI + INSTI	2 (6)	4 (11)	6 (9)	…
bPI + INSTI	5 (14)	2 (6)	7 (10)	…
NNRTI + INSTI	2 (6)	3 (9)	5 (7)	…
bPI + INSTI + MVC	0 (0)	3 (9)	3 (4)	…

Data are presented as median (IQR) or No. (%).

Abbreviations: BL, baseline; bPI, boosted protease inhibitor; FTR, fostemsavir; IBA, ibalizumab; INSTI, integrase strand transfer inhibitor; MVC, maraviroc; NNRTI, nonnucleoside reverse transcriptase inhibitor; NRTI, nucleoside reverse transcriptase inhibitor; VL, viral load.

^a^Prior regimen received by at least 3 participants in the FTR and IBA groups.

### Baseline Virologic Characteristics

**Figure 1.**
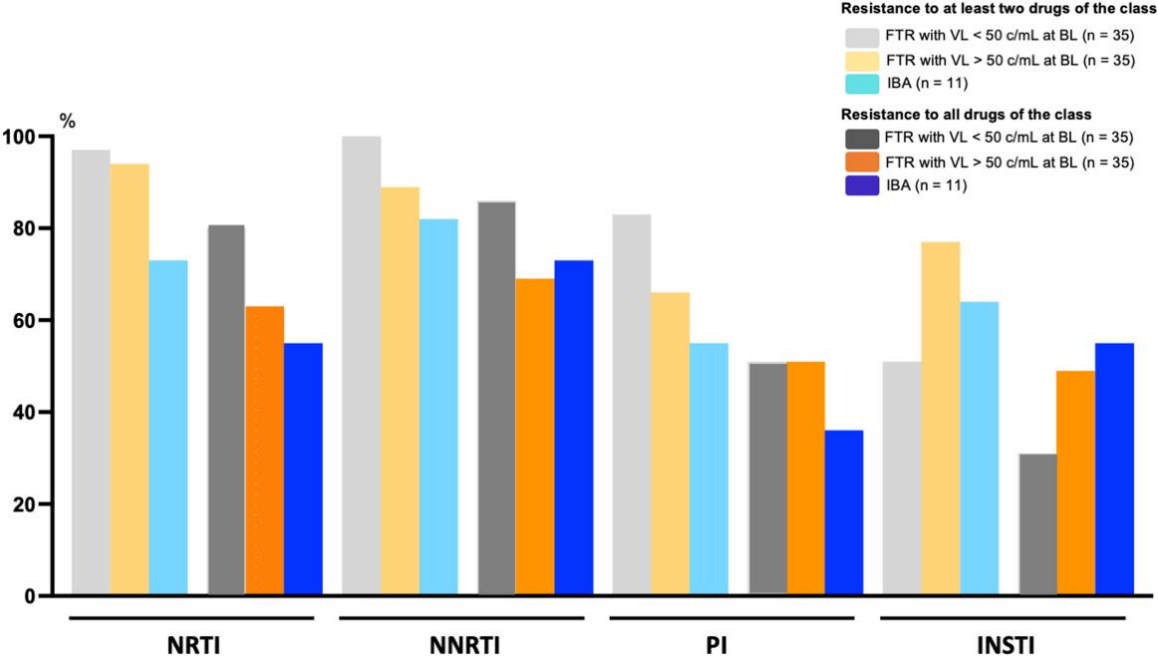
Percentage of participants with viruses resistant to at least 2 drugs of an antiretroviral drug class or resistant to all drugs of the antiretroviral drug class. BL, baseline; FTR, fostemsavir; IBA, ibalizumab; INSTI, integrase strand transfer inhibitor; NNRTI, nonnucleoside reverse transcriptase inhibitor; NRTI, nucleoside reverse transcriptase inhibitor; PI, protease inhibitor; VL, viral load.

The participants had a median 4 and 6 historical GRTs at the initiation of FTR- and IBA-based treatment, respectively. Among all PWH receiving FTR, the mean numbers of drug resistance mutations were 6 (range, 0–9), 4 (0–7), 4 (0–8), and 2 (0–5) for NRTI, nonnucleoside reverse transcriptase inhibitor (NNRTI), PI, and INSTI (Table 2). The percentages of participants having a virus resistant to at least 2 drugs of the class were 96%, 94%, 74%, and 64% for NRTI, NNRTI, PI, and INSTI (Figure 1). The percentages of participants receiving FTR having a virus resistant to all drugs of the class were 71%, 77%, 51%, and 40% for NRTI, NNRTI, PI, and INSTI.

**Table 2.** Number of Historical Resistance Genotypes and Cumulative Drug Resistance Mutations

	FTR (n = 70)			
	VL <50 Copies/mL at BL (n = 35)	VL >50 Copies/mL at BL (n = 35)	All (n = 70)	IBA (n = 11)
Historical resistance genotypes	5 (3–8)	4 (2–7)	4 (2–8)	6 (4–9)
Resistance mutations				
NRTI	7 (5–7)	6 (3–8)	6 (5–7)	5 (2–8)
NNRTI	4 (3–5)	3 (2–4)	4 (3–5)	3 (2–4)
Major PI	4 (3–5)	4 (0–5)	4 (1–5)	3 (0–6)
INSTI	1 (0–2)	2 (1–4)	1 (0–3)	1 (1–4)

Data are presented as median (IQR).

Abbreviations: BL, baseline; FTR, fostemsavir; IBA, ibalizumab; INSTI, integrase strand transfer inhibitor; NNRTI, nonnucleoside reverse transcriptase inhibitor; NRTI, nucleoside reverse transcriptase inhibitor; PI, protease inhibitor; VL, viral load.

Among PWH receiving IBA, the mean numbers of drug resistance mutations were 4 (range, 0–9), 3 (0–7), 3 (0–7), and 2 (0–6) for NRTI, NNRTI, PI, and INSTI, respectively (Table 2). The percentages of participants having a virus resistant to at least 2 drugs of the class were 73%, 82%, 55%, and 64% for NRTI, NNRTI, PI, and INSTI (Figure 1). The percentages of participants receiving IBA having a virus resistant to all drugs of the class were 55%, 73%, 36%, and 55% for NRTI, NNRTI, PI, and INSTI.

Overall, among the 81 participants of this study, the most common resistance mutations were M184V (n = 68, 84%), Y181C/I/V (n = 45, 56%), M46I/L (n = 51, 63%), and Q148H/K/R (n = 25, 31%) for NRTI, NNRTI, PI, and INSTI, respectively (Supplementary Figure 1).

*Env* sequencing before initiation of FTR-based treatment was available for 25 PWH showing a polymorphism associated with FTR resistance in 10 cases: S375I/T (n = 9), M426L (n = 1), and M434K (n = 2), with viruses harboring 2 polymorphisms in 2 cases.

### OBT and Pharmacovirologic Outcome Among Participants Receiving FTR

**Figure 2.**
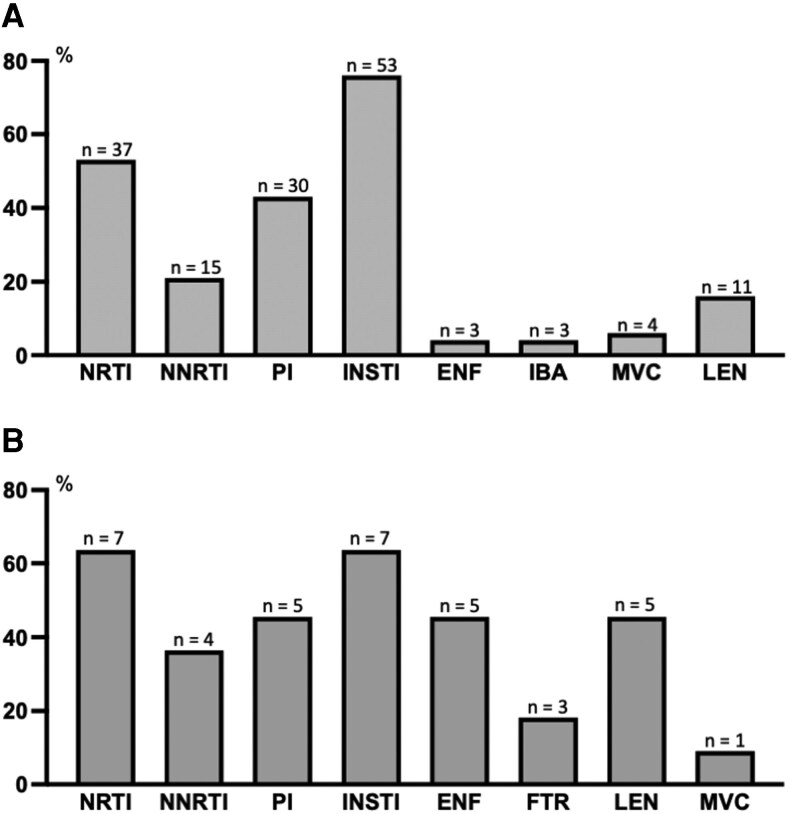
Description of the major antiretroviral drug classes and entry inhibitors included in the optimized background therapy added to fostemsavir (*A*) or ibalizumab (*B*). ENF, enfuvirtide; FTR, fostemsavir; IBA, ibalizumab; INSTI, integrase strand transfer inhibitor; LEN, lenacapavir; MVC, maraviroc; NNRTI, nonnucleoside reverse transcriptase inhibitor; NRTI, nucleoside reverse transcriptase inhibitor; PI, protease inhibitor.

**Figure 3.**
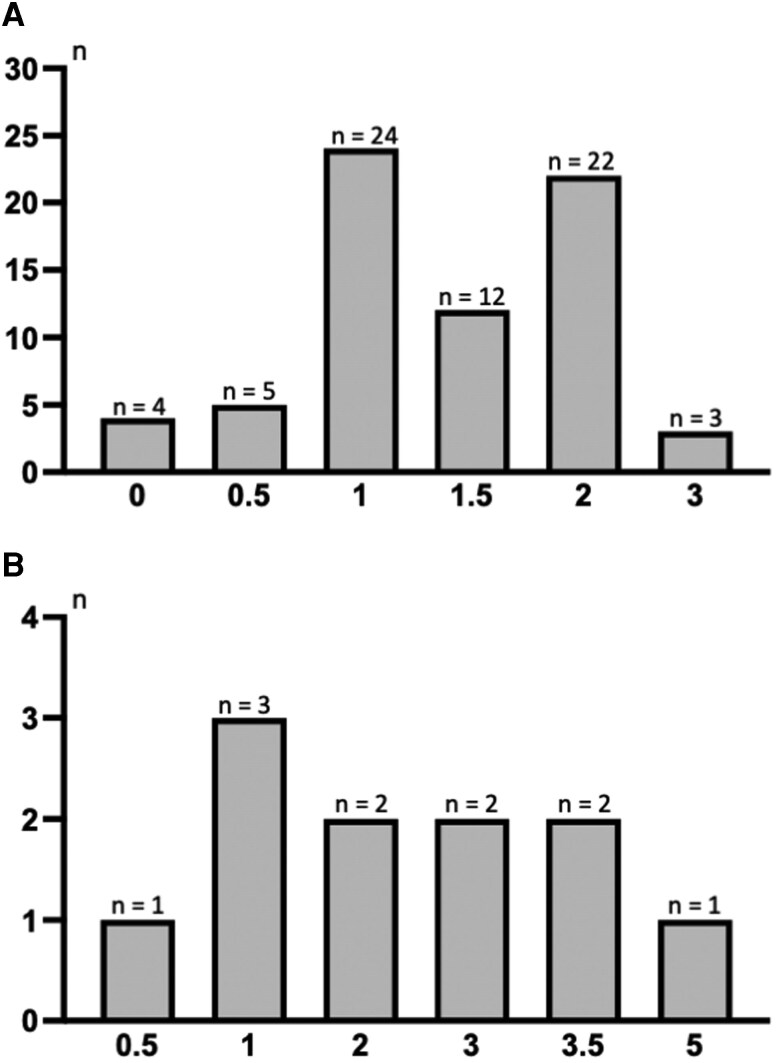
Distribution of the genotypic susceptibility score for fostemsavir study (*A*) or ibalizumab study (*B*).

The median number of ARVs in the OBT was 2 (IQR, 2–3), with the INSTI drug class being the most frequently used (76% of cases; Figure 2). Eleven individuals (16%) were also receiving the capsid inhibitor LEN. Overall, the GSS, based on cumulative historical genotypes, was ≤1 in 33 participants (47%) from which 4 had no active drugs in the OBT (Figure 3).

The median follow-up time of FTR-based treatment was 20 months (IQR, 12–37). During the follow-up, 8 individuals experienced a VF (11%), and 8 had a profile of nonvirologic response (11%). The median VL was 157 copies/mL (IQR, 105–115; range, 55–47 000) at the first time point of VF and 950 copies/mL (IQR, 155–11 083; range, 120–30 000) at the last time point for the 8 nonresponders.

At the last follow-up visit, 54 (78%) participants had a VL <50 copies/mL, reaching 57 (82%) at the threshold of 200 copies/mL (Table 3). Regarding the virologic outcome, depending on the baseline VL, virologic suppression was maintained in 32 (91%) participants who were aviremic at baseline, and it was obtained in 22 (63%) participants in failure at initiation. There was no difference in the median GSS between participants in virologic success and those in VF, equal to 1.5 in both groups. No association was observed between the presence of baseline *env* polymorphisms and the virologic outcome. Indeed, among the 10 participants harboring *env* polymorphisms, 7 had a VL <50 copies/mL at the last follow-up visit.

**Table 3.** Virologic Outcome at Last Follow-Up Visit, Depending on HIV Viral Load at Initiation, at the Virologic Suppression Thresholds of 50 and 200 Copies/mL

s	FTR (n = 70)			
Threshold: Virologic Outcome at Last Follow-Up Visit	VL <50 Copies/mL at BL (n = 35)	VL >50 Copies/mL at BL (n = 35)	All (n = 70)	IBA (n = 11)
50 copies/mL				
Virologic success	32 (91)	22 (63)	54 (78)	4 (36)
Virologic failure	3 (9)	5 (14)	8 (11)	5 (46)
Nonresponse	…	8 (23)	8 (11)	2 (18)
200 copies /mL				
Virologic success	35 (100)	22 (63)	57 (82)	4 (36)
Virologic failure	…	5 (14)	5 (7)	5 (46)
Nonresponse	…	8 (23)	8 (11)	2 (18)

Data are presented as No. (%).

Abbreviations: FTR, fostemsavir; IBA, ibalizumab; VL, viral load.

The median increase in CD4 cell count at the last follow-up visit was +118/mm^3^ (IQR, 40–367) for PWH in treatment failure at initiation and +34/mm^3^ (IQR, −44 to 113) for participants in virologic suppression at initiation.

Treatment discontinuation was observed in 20 participants: 7 for VF, 5 for simplification, 3 for deaths unrelated to FTR, 2 for nonadherence, 2 for loss to follow-up, and 1 for drug-drug interactions.

A GRT result was available for 8 PWH, 4 in VF and 4 nonresponders, showing the emergence of gp120 FTR resistance mutations in 1 case: S375N and M426L. Emergence of new drug resistance mutations to the associated ARVs was observed in 1 case with the K65R and M184 V mutations. Plasma levels of temsavir were available for 7 PWH (5 in nonresponders and 2 in VF), including 4 receiving a boosted PI, with a median level of 1252 ng/mL. Suboptimal temsavir plasma concentrations (ie, <200 ng/mL) were observed in 2 cases, both of which were in VF and not receiving a boosted PI. Plasma levels of the ARV drugs included in the OBT were available for the same 7 participants, showing that those with suboptimal temsavir levels also had suboptimal concentrations of their OBT.

Regarding the 11 participants who received LEN in the OBT, 9 (82%) had VLs <50 copies/mL at the last follow-up visit and 2 were nonresponders.

### Virologic Outcome Among Participants Receiving IBA

The median number of ARVs in the OBT was 4 (range, 2–7), with the NRTI and INSTI drug classes being the most frequently used (64% of cases; Figure 2). Since the participants were HTE with MDR viruses, 5 were also receiving LEN, 5 the fusion inhibitor enfuvirtide, and 3 FTR. Overall, the GSS, based on cumulative historical genotypes, was ≤1 in 4 participants (36%; Figure 3).

The median follow-up was 7 months (IQR, 3–43). IBA-based treatment was discontinued for 8 participants (73%): 2 for deaths unrelated to IBA, 3 for simplification, 2 for follow-up loss, and 1 for VF. At the last follow-up visit, 4 participants had VLs <50 copies/mL, 2 were nonresponders, and 5 experienced a VF with a median VL of 293 copies/mL (IQR, 265–868; range, 243–47 000). A GRT result was available for 4 PWH with the emergence of the N74D in the capsid, a new drug resistance mutation to the associated ARVs in 1. Suboptimal concentrations of associated ARVs were observed in 3 of the 5 assessable participants. Differential adherence between injectable and oral medications was observed, as only 1 PWH had adequate concentrations of oral ARVs.

## DISCUSSION

In this study, we report the outcomes in real-world settings of PWH initiating FTR or IBA: 2 entry inhibitors that are used in the HTE population harboring MDR viruses. Virologic success was maintained in 91% of PWH who were virologically suppressed and initiating an FTR-based treatment, and success was respectively achieved in 60% and 36% of PWH receiving failing therapy with detectable VLs at FTR- or IBA-based treatment initiation.

This study was one of the first to assess FTR use and clinical outcomes in a real-world setting outside the United States, where it was first approved in 2020. Most of our knowledge on the clinical efficacy of this molecule comes from the BRIGHTE trial, which enrolled only individuals with VLs ≥400 copies/mL and drug resistance [16, 17, 19, 20]. Similar to our study, the OPERA cohort, a large database representative of routine HIV clinical care in the United States, conducted an observational study reflecting real-world clinical practices, enrolling all participants initiating FTR, including those who were virologically suppressed [30]. One important difference among the 3 studies is the definition of VF. Here it was defined as 2 consecutive plasma VLs ≥50 copies/mL, while it was 200 copies/mL in the OPERA study and 400 copies/mL in the BRIGHTE trial. We also defined a virologic nonresponse as a VL decrease <1 log_10_ copies/mL or failure to achieve virologic suppression (<50 copies/mL) at week 24.

In the present study, within a median 20 months of follow-up of FTR-based treatment, 62% of PWH who were viremic achieved viral suppression, which was very similar to the response rate of the BRIGHTE trial in the randomized cohort at week 96 (60%) [16]. This was slightly higher than among the viremic/low CD4 group in OPERA (31% at 6 months, 33% at 12 months) [30]. The baseline CD4 T-cell counts was lower in OPERA as compared with our study participants (median, 110 vs 219 cells/mm^3^), which could suggest a more advanced level of HTE in OPERA, but this was also the case among BRIGHTE trial participants. These findings may further be influenced by variations in the definitions used to classify PWH with MDR as HTE. For instance, the OPERA study included all PWH receiving FTR, irrespective of their resistance profile. Consequently, neither the overall resistance burden nor the number of active agents included in the OBT was reported. Importantly, 53% of our study participants and 40% of the randomized cohort of BRIGHTE had >1 active drug in the OBT [17]. In line with this observation, a recent follow-up analysis of the BRIGHTE study demonstrated that a lower number of fully active available ARVs in OBT and a lower overall susceptibility score to newly used ARVs in OBT were associated with lower odds of virologic response at week 96 [19]. It is important to note that the overall susceptibility score is based on a combination of genotypic and phenotypic susceptibility assessments, whereas the GSS alone may not adequately predict long-term virologic responses [19, 31]. Accordingly, in our study, the median GSS did not differ between participants with virologic success and those with VF. In addition, the INSTI drug class was the most frequently used in the OBT in our study (76% of cases), and it has been shown that the presence of dolutegravir in the OBT was associated to virologic response through week 192 [19]. Optimization of the background agents taken with FTR is critical in HTE cases, and physicians should consider using second-generation INSTI as part of the regimen. By the end of follow-up in our study, this new FTR-based regimen led to an increase of CD4+ T-cell counts (median, 118 cells/mm^3^), confirming in real life the potential benefit of FTR as a treatment option for this vulnerable population.

The pivotal trial of FTR that enabled its approval in clinic focused on people who were HTE and experiencing treatment failure, enrolling almost exclusively participants with detectable VLs [17]. An interesting finding of the real-world observational studies is that a third of the participants in OPERA to half of those in the present study were actually virologically suppressed before FTR initiation [30]. The main difference in the population characteristics was the mean CD4 T-cell count at FTR initiation, which was twice as high for participants who were aviremic and >500 cells/mm^3^ (median, 585 vs 219 cells/mm^3^). This could indicate that the group of participants experiencing VF has more advanced disease and more adherence problems. The number of historical resistance genotypes and drug resistance mutations within each drug class was similar in the 2 populations, however, ranging from 2 to 6. This highlights the fact that the participants with virologic suppression at FTR initiation were also representative of people who were HTE and had MDR viruses. These results suggested that FTR might be useful in this situation, showing very good clinical efficacy, with 91% of virologic success after 20 months of treatment at the threshold of <50 copies/mL and 100% at <200 copies/mL. The reasons for changing the drug regimen and using FTR were manyfold, including tolerance, safety considerations, drug-drug interaction, or simplification. In this study, we were unable to assess the specific reasons for FTR initiation. Yet, it is worth noting that the use of PIs dropped from 71% to 20% between the previous regimen and the OBT added to FTR. Furthermore, enfuvirtide was no longer used, which suggests concerns about long-term tolerance and the need to optimize treatment. FTR was well tolerated in phase 2b and 3 trials [16, 32], although there is no direct comparison against specific ARVs (eg, boosted darunavir). Furthermore, there is no large-scale and long-term safety profile, which will be important to consider in the future.

In Italy, the PRESTIGIO Registry is a real-world evidence study assessing the long-term efficacy of different ARV regimens in PWH with resistant viruses to 4 drug classes. It recently reported the 1-year outcome of 52 participants initiating an FTR-containing regimen, including 40 who were viremic and 12 nonviremic [33]. After a median follow-up of 15.6 months (IQR, 9.0–31.3), 11 (21%) discontinued FTR, with the most frequent cause of discontinuation being VF. The researchers estimated the probability of treatment retention at 4 years to be 67.4% (95% CI, 42.6%–83.3%) and the probability of virologic success at 2 years to be 80%. However, there are no studies with a longer follow-up period than ours to support these data. It will be important to evaluate the long-term efficacy of FTR-based regimens.

IBA was the first monoclonal antibody approved for the treatment of HIV-1 infection, but the available data on its clinical efficacy remain scarce and are limited to studies involving few participants [11, 34–37]. The pivotal study enrolled only 40 participants, with 31 completing it. At week 24, 43% of the patients had a VL <50 copies/mL [11]. More recently, a follow-up expanded-access phase 3 study evaluated the efficacy of IBA plus an OBT in PWH who were HTE [35]. In 38 participants who were IBA naive, experiencing VF, and receiving compassionate access, 11 of 24 (46%) and 7 of 18 (47%) had a VL <50 copies/mL at weeks 24 and 48, respectively. Another arm of this study enrolled participants with prior exposure to IBA, ranging from 24 weeks to 6.9 years. Among them, 23 of 39 (59%) had a VL <50 copies/mL at study initiation, and 27 of 35 (77%) achieved or maintained suppressed viremia at week 24 and 24 of 33 (72.7%) at week 48. The long-term efficacy and experience of using this molecule are limited to a very small number of participants who are willing to continue a regimen that includes IBA. In France and Europe, the commercial availability of IBA was stopped in 2022. In our study, we were able to enroll only 11 participants with at least 3 months of follow-up in our national multicenter study. The median follow-up was 7 months (IQR, 3–43), and the reasons for discontinuation were simplification, loss of follow-up, death, or VF. The proportion of virologic success was 36%, which was similar to the previous studies [11, 34, 37]. Of note, 5 individuals were also receiving LEN, as they were HTE with MDR viruses, which may be a confounding factor when interpreting clinical efficacy. Combining IBA and LEN with OBT may be beneficial for this specific population of PWH, as a recent case series of 21 individuals showed clinically significant reductions in VL and improvements in CD4 cell counts [34].

This retrospective observational study reflected real-world clinical practices and was representative of routine HIV clinical care. However, there were several limitations, including the absence of a comparator group and longitudinal sampling, the lack of clinical data, as well as incomplete data regarding resistance at failure. Thus, we were not able to evaluate the immunologic response to IBA-based regimen change due to incomplete CD4 data and the low number of participants. Yet, in people who were HTE with CD4 cell counts <100/mm^3^, initiating an IBA-based regimen with optimized OBT was associated viral suppression and a significant increase in CD4 cell count [11]. There were also limited data regarding GRT results and drug concentrations. Among the 8 participants experiencing VF and the 8 nonresponders to the new FTR-based regimen, data were available for half of them, and emergence of FTR resistance mutations could be identified in only 1 case (S375N and M426L). Resistance mutation selection to the OBT was observed in a second case, as well as in a third case receiving IBA. Suboptimal levels of temsavir and the OBT were measured in both cases of VF for which drug concentrations were available, suggesting a drug adherence problem. This was also the case for oral ARVs associated with IBA in 3 of 5 participants with available drug concentrations. In people who were HTE with limited treatment options, complex individualized regimens may lack the simplicity and tolerability of first-line single-tablet regimens, thereby increasing the risk of poor adherence [1–3]. In this context, adherence support interventions are strongly recommended, as emphasized in the World Health Organization 2025 guidelines [38]. In addition, therapeutic drug monitoring may help identify suboptimal adherence. Nevertheless, treatment failure is often multifactorial and usually difficult to explain in these diverse cohorts. For example, 5 FTR nonresponders for whom plasma drug concentrations were available had drug levels within the therapeutic range. The impact of baseline and on-treatment variables on the durability of responses to FTR in the BRIGHTE study has been extensively explored. In the randomized cohort, VF risk at week 96 did not differ according to the presence of predefined gp120 amino acid substitutions with potential impact on phenotypic susceptibility to temsavir or according to baseline temsavir 50% inhibitory concentration fold change [20]. Although the dataset was more limited in our study, no association was observed between the presence of baseline *env* polymorphisms and the virologic outcome. FTR and IBA are entry inhibitors that block the interaction between the gp120 and the receptor CD4. This interaction and the structure of the envelope trimer of HIV are much more complex than the enzymes targeted by previous ARVs, and resistance pathways are more complex to understand and predict [26, 27, 39]. This is also the case for broadly neutralizing antibodies targeting the CD4-binding site, where temsavir binds, for which the correlation with genotypic prediction, in vitro susceptibility, and clinical efficacy is still poorly understood [40, 41]. The impact of individual substitutions is influenced by the broader gp120 sequence and structural background [42]. Further studies and prospective surveillance are required to accumulate data, particularly from the whole envelope, with sequences before and at the time of failure. Overall, it is very unlikely that standardized resistance tests for FTR and IBA that accurately predict their clinical efficacy will be available soon. In this context, advances in generative artificial intelligence could be useful, provided that we have a sufficiently large database containing *env* sequences, in vitro neutralization data, and clinical outcome.

In conclusion, this real-life retrospective study showed that PWH receiving FTR- or IBA-based treatment harbored MDR viruses and had very limited treatment options. Among PWH undergoing failing regimens, virologic success was achieved in 60% and 36% with detectable VLs at FTR- or IBA-based treatment initiation, respectively, confirming the potential benefit of these new treatment options for this vulnerable population. People who are HTE and virologically suppressed maintain virologic control after 2 years to switching to an FTR-based regimen, which represents a valuable option for treatment optimization.

## Supplementary Material

ofag495_Supplementary_Data

## Supplementary Data

Supplementary materials are available at *Open Forum Infectious Diseases* online. Consisting of data provided by the authors to benefit the reader, the posted materials are not copyedited and are the sole responsibility of the authors, so questions or comments should be addressed to the corresponding author.
